# Dose-response relationship among body mass index, abdominal adiposity and atrial fibrillation in patients undergoing cardiac surgery: a meta-analysis of 35 cohorts

**DOI:** 10.7717/peerj.11855

**Published:** 2021-07-21

**Authors:** Menglu Liu, Kaibo Mei, Lixia Xie, Jianyong Ma, Peng Yu, Siquan Niu, Ya Xu, Yujie Zhao, Xiao Liu

**Affiliations:** 1 Department of Cardiology, Seventh People’s Hospital of Zhengzhou, Zhengzhou, Henan, China; 2 Anesthesiology Department,, the People’s Hospital of Shanggrao, Shangrao, Jiangxi, China; 3 Department of Respiratory and Critical Care Medicine, the Second Affiliated Hospital of Nanchang University, Nanchang, Jiangxi, China; 4 Department of Pharmacology and Systems Physiology, University of Cincinnati College of Medicine, Cincinnati, Oh, China; 5 Department of Endocrine, the Second Affiliated Hospital of Nanchang University, Nanchang, Jiangxi, China; 6 Department of Cardiology, Sun Yat-sen Memorial Hospital of Sun Yat-sen University, Guangzhou, Guangdong, China; 7 Guangdong Province Key Laboratory of Arrhythmia and Electrophysiology, Guangzhou, Guangdong, China; 8 Guangzhou Key Laboratory of Molecular Mechanism and Translation in Major Cardiovascular Disease, Guangzhou, China

**Keywords:** Atrial fibrillation, Body mass index, Risk factor, Meta-analysis

## Abstract

**Background:**

Whether overweight increases the risk of postoperative atrial fibrillation (POAF) is unclear, and whether adiposity independently contributes to POAF has not been comprehensively studied. Thus, we conducted a meta-analysis to clarify the strength and shape of the exposure-effect relationship between adiposity and POAF.

**Methods:**

The PubMed, Cochrane Library, and EMBASE databases were searched for revelant studies (randomized controlled trials (RCTs), cohort studies, and nest-case control studies) reporting data regarding the relationship between adiposity and the risk of POAF.

**Results:**

Thirty-five publications involving 33,271 cases/141,442 patients were included. Analysis of categorical variables showed that obesity (RR: 1.39, 95% CI [1.21–1.61]; *P* < 0.001), but not being underweight (RR: 1.44, 95% CI [0.90–2.30]; *P* = 0.13) or being overweight (RR: 1.03, 95% CI [0.95–1.11]; *P* = 0.48) was associated with an increased risk of POAF. In the exposure-effect analysis (BMI) was 1.09 (95% CI [1.05–1.12]; *P* < 0.001) for the risk of POAF. There was a significant linear relationship between BMI and POAF (P_nonlinearity_ = 0.44); the curve was flat and began to rise steeply at a BMI of approximately 30. Notably, BMI levels below 30 (overweight) were not associated with a higher risk of POAF. Additionally, waist obesity or visceral adiposity index was associated with the risk of POAF.

**Conclusion:**

Based on the current evidence, our findings showed that high body mass index or abdominal adiposity was independently associated with an increased risk of POAF, while underweight or overweight might not significantly increase the POAF risk.

## Introduction

The prevalence of overweight and obesity has rapidly increased in recent decades in both developing and developed counties (Jayedi et al., 2018; Milajerdi et al., 2019; Vogli et al., 2014). This increase has raised serious public health concerns due to the positive association between overweight and obesity and an increased risk of various chronic diseases, including cardiovascular diseases, metabolic syndrome, all-cause mortality, and several types of cancer (Aune et al., 2016; Guh et al., 2009; Hennessy et al., 2019; Rahmani et al., 2019).

Postoperative atrial fibrillation (POAF) is among the most common complications arising from cardiac surgery, affecting between 20% and 40% of patients undergoing cardiac operation (Almassi et al., 1997; Dobrev et al., 2019; Mariscalco & Engström, 2009), and is associated with significantly worse adverse outcomes (such as all-cause death and stroke) (Mitchell & Committee, 2011). Several cohort studies have reported increased POAF with a higher body mass index (Bramer et al., 2011; Engelman et al., 1999; Melduni et al., 2011) (BMI). Although various studies reported a positive association between obesity (BMI > 30 kg/m^2^) and POAF in patients undergoing cardiac surgery, whether being overweight (BMI of 25–29.9 kg/m^2^) increases the risk remains unclear as some studies found a positive association (Yap et al., 2007; Zacharias et al., 2005), while other studies did not (Echahidi et al., 2007; Girerd et al., 2009; Reeves et al., 2003). Several well-designed meta-analyses based on a categorical or continuous model found an increased risk of POAF among individuals with obesity. These studies provide valuable information. However, these studies have several limitations. First, it is arguable that the use of a categorical model in meta-analyses has the risk of reducing power and precision by dividing the exposure into several groups (Bennette & Vickers, 2012). Furthermore, the use of a continuous model might not detect the dose-specific association as several studies have also reported a “U”-shaped association between BMI and POAF (Girerd et al., 2009; Sun et al., 2011). Second, some important factors associated with the risk of AF, such as chronic obstructive pulmonary disease (COPD), smoking, diabetes, and left atrial diameter (LAD), were reported to have a higher prevalence in individuals with obesity and those undergoing cardiac surgery. Nevertheless, whether obesity independently increases the risk of POAF is still unclear according to meta-analyses. For example, in an excellent comprehensive review involving 62,160 individuals and 16,768 cases (Wong et al., 2015a), BMI was found to be an independent risk factor for POAF; however, the same result for BMI was not found by recent meta-analysis (Yamashita et al., 2019) or literature reviews (Higgs, Sim & Traynor, 2020). Third, to date, no comprehensive study has quantitatively assessed the exposure-effect relationship between BMI and POAF. The shape of the exposure–effect curve and whether being overweight independently increases the risk of POAF are still unclear. Moreover, several studies have also assessed the association between abdominal measures of adiposity, such as waist circumference, and advocated that these measures are a better index for predicting the incidence of POAF (Gao et al., 2016; Girerd et al., 2009). Clarifying the dose–response relationship may help to elucidate whether there are any threshold effects between BMI or abdominal adiposity and the risk of POAF, which would be of major importance from a public health perspective and for improving guidelines to manage risk factors for primary prevention.

Thus, we conducted a dose–response meta-analysis to quantify the association among body mass index, abdominal adiposity and the incidence of POAF.

## Methods

This study was performed according to Preferred Reporting Items for Systematic reviews and Meta-Analyses Statement (PRISMA) guidelines (Moher et al., 2009) (Table S1). All studies, including RCTs and observational studies (cohort and nested case-control studies), reporting data regarding BMI and POAF were considered eligible for this systematic review. A systematic literature search was conducted using the Cochrane Library, PubMed, and EMBASE databases through December 2019. Two researchers (X.L. and M.L-L) independently performed the entire process of this exposure-effect meta-analysis from the literature search and selection to the data analysis. Table S2 provides a detailed description of the search strategy. All discrepancies were resolved through discussion with each other or consultation with a 3rd reviewer (K.B-M). We used the method described by Greenland and Longnecker (Greenland, 1987) to estimate the study-specific linear trends and 95% CIs from the natural logs of the RRs (risk ratio) and CIs across categories of BMI. The robust error meta-regression method was used to fit the nonlinear exposure-effect meta-analysis of BMI and POAF (Doi & Sar, 0000; Xu et al., 2018; Zhang et al., 2018). All statistical analyses were performed using Review Manager (RevMan) version 5.3 (The Cochrane Collaboration 2014; Nordic Cochrane Center Copenhagen, Denmark) and Stata software (Version 14.0, Stata Corp LP, College Station, Texas, USA). *P* < 0.05 was considered statistically significant. The full details of the literature search strategy, study selection criteria, quality assessment, and statistical analysis are reported in the Supplemental Methods. Additionally, this study was registered with PROSPERO (international prospective register of systematic reviews) under registration number CRD42019128770.

## Results

### Study selection

**Figure 1:**
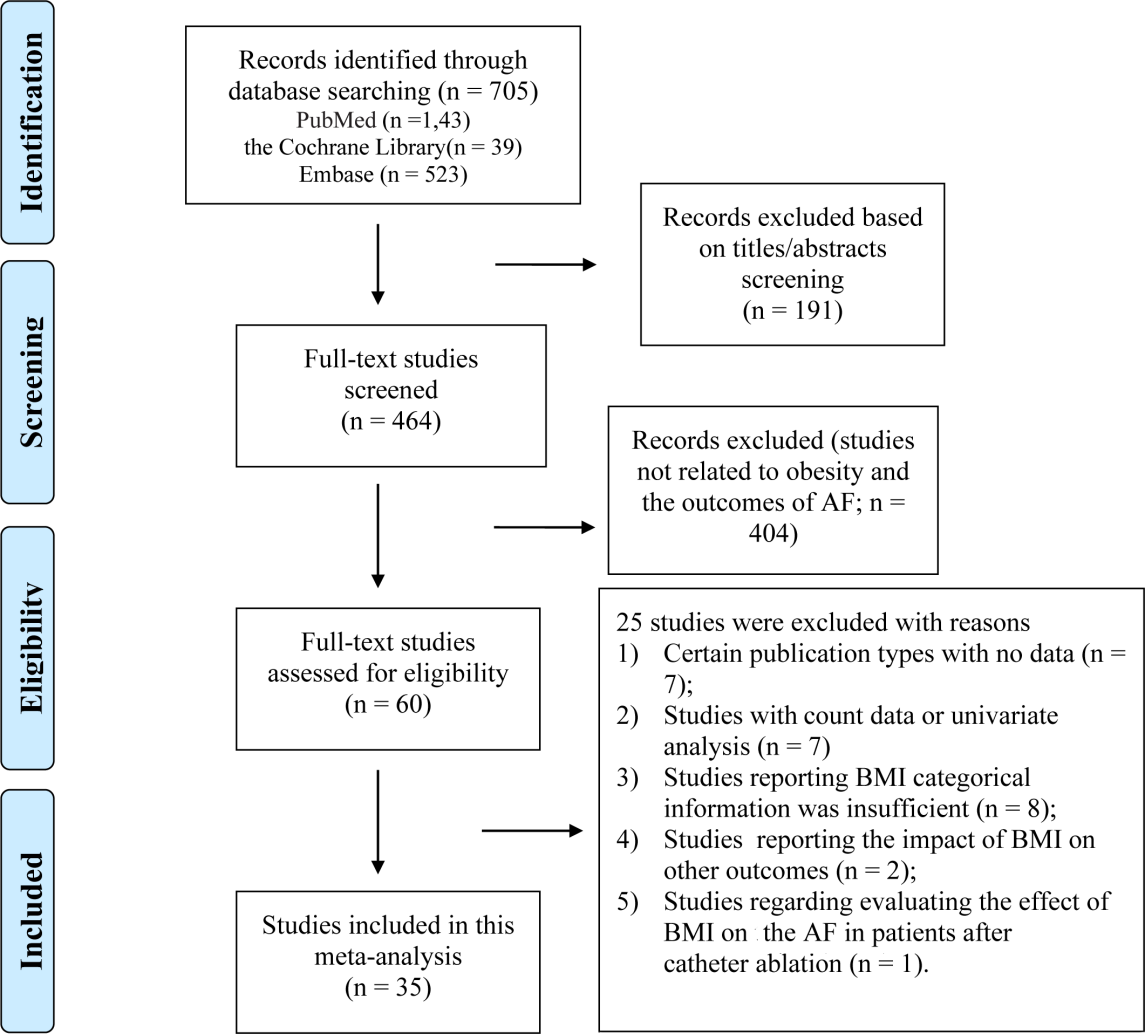
Overview of the research strategy. RR, risk ratio.

As shown in Fig. 1, a total of 705 articles were initially identified, including 189 duplicates. After screening the titles and abstracts, 60 articles remained for the detailed full-text screening. All articles excluded after the full-text review are listed in Table S3 in the Supplemental Material. Finally, a total of 34 studies (35 cohorts) were included. As shown in Table 1, thirty-one (32 cohorts) studies were included in the analysis of BMI, and two studies were included in the analysis of waist circumference, two studies examined the BMI and waist circumference both, and one study was included in the analysis of visceral adiposity index. Twenty (21 cohorts) articles reported AF after CABG, three articles reported AF after valve surgery, and eleven articles reported the total AF after various cardiac surgeries.

**Table 1:** Basic characteristics of the 35 cohorts included in the meta-analysis.

**Author, publication year, country**	**Source of participants**	**Cases/N**	**Mean age (years), male (%)**	**Study design**	**Method of AF Detection**	**BMI Data Reported**	**Type of operation**	**Adjustment for confounders**
Alam, 2011, USA	St. Luke’s Episcopal Hospital/Texas Heart Institute	2867/13115	63.3, 54.4	Retrospective cohort study	ECG, treating physician	<30 ≥30	CABG	Age, sex, preoperative morbidity, extent of CAD, No. of CABG, use of internal mammary artery, total circulatory bypass time, ACT.
Bramer, 2011, Netherlands	Catharina Hospital	2517/9348	64.2, 72.6	Prospective cohort study	ECG,	Continuous variable	CABG or valve surgery	Age, BSA, COPD, PVD, prior stroke, prior MI, LVEF, creatinine, type of procedure, ECC duration, transfusion of RBCs, FFP and platelets, and reoperation for bleeding.
Brandt, 2001, Germany	University Hospital Kiel	207/500	63, 82	Retrospective cohort study	NA	<30 ≥30	CABG	Sex, history of prior MI, COPD, previous stroke, duration of CPB, ACT and number of distal anastomoses performed.
Banach (aortic stenosis), 2007, Poland	Department of Cardiac Surgery in Lodz	62/150	63.3, 48.7	Retrospective cohort study	ECG,	≤21 ≥30	valve surgery	Age, BMI, pre-operative and post- operative LVEF, mitral regurgitation.
Engelman, 1999, USA	Brigham and Women’s Hospital	1518/5168	67, 68	Retrospective cohort study	NA	<20 20–30 >30	CABG or valve surgery	Age, sex, EF, NYHA functional class, previous cardiac operation, pre-operative diabetes, peripheral and cerebral vascular disease, hypertension, renal failure, CHF, MI, COPD, smoking, urgency of operation, use of an ITA, and type of operation.
Bidar, 2014, Netherlands	Maastricht University Medical Centre	73/148	67.1,80.6	Prospective cohort study	ECG	Continuous variable	CABG or valve surgery	Sex, DM, baseline CRP, smoke, early POAF, aortic clamp time, creatinine levels
Echahidi, 2014, Canada	The Quebec Heart Institute	1370/5086	64.1, 76.5	Retrospective cohort study	ECG	<25 25–30 30–35 ≥35 Continuous variable	CABG	Age, gender, BMI, DM, left main coronary stenosis, preoperative medication with *β*-blockers, and ACT.
Engin, 2020, Turkey	Bursa Yüksek İhtisas Training and Research Hospital	55/199	58.2, 80.4	Prospective cohort study	ECG	Continuous variable (Visceral Adiposity Index)	CABG	Age, Hypertension, COPD, Triglyceride Lymphocyte, CRP, prognostic nutritional index;
El-Chami, 2012, USA	Emory University Hospital or Emory Crawford Long Hospital	3486/18517	62.5, 71.7	Retrospective cohort study	NA	NA	CABG	Age, race, gender, height, weight, BMI, body surface area, last creatinine level, angina, left main CAD, immunosuppressive therapy, preoperative insertion of an intra-aortic balloon pump, number of diseased vessels and preexisting medical conditions.
Erdil N, 2013, Turkey	Inonu University, School of Medicine	129/1040	60.2, 75.8	Retrospective cohort study	ECG, physician assessment	Continuous variable	CABG	Age, additive EuroSCORE score, and prolonged ventilation.
Efird (Black), 2016, USA	East Carolina Heart Institute	376/2329	NA, 58.1	Retrospective cohort study	Medical record	<18.5 18.5–25 25–30 30- 35 35- 40 ≥40	CABG	Age, sex, DM, unstable heart failure, hypertension, PAD, three-vessel disease.
Efird (White), 2016, USA	East Carolina Heart Institute	2627/11265	NA, 72.8	Retrospective cohort study	Medical record	<18.5 18.5–25 25–30 30–35 35–40 ≥40	CABG	Age, sex, DM, Unstable heart failure, hypertension, PAD, Three-vessel disease.
Gao, 2016, China	First Affiliated Hospital of Nanjing Medical University	1183/4740	63.6, 68.7	Retrospective cohort study	NA	<18.5 18.5–25 25–30 30–35 35–40 ≥40	Valve surgery	Age, gender, surgery type, family history of CAD, diabetes, hypertension, heart failure and lipid lowering medication.
Ghanta, 2017, USA	Regional Society of Thoracic Surgeons certified database	3052/13637	65.6, 70.7	Retrospective cohort study	Medical records	18.5–30 30 ≤ BMI ≤40 >40	CABG and/or valve surgery	STS PROM, age, sex, presence of hypertension, DM, renal failure, and heart failure.
Girerd, 2009, Canada	Quebec Heart Institute	433/2214	56.2, 100	Nested case–control	ECG	<25 25–30 30–35 35–40 ≥40	CABG	Waist circumference and age.
Gürbüz, 2014, Turkey	Medicana International Ankara Hospital	139/790	62, 77.8	Retrospective cohort study	ECG	≤ 30 >30	CABG	Age, sex, DM, hypertension, hyperlipidemia, preoperative arrhythmia and atrial fibrillation, PAD, history of cerebrovascular disease, preoperative echocardiography data, history of clopidogrel use, operation type, CPB and cross clamp times, number of grafts, extubation time, intensive care unit and hospital length of stay times, amount of drainage, number of used blood and blood products, postoperative creatinine and creatinine kinase levels, occurrence of postoperative arrhythmia and stroke.
Hakala, 2002, Finland	Kuopio University Hospital	30/92	61.7, 76	Prospective cohort study	ECG	Continuous variable	CABG	Age, preoperative haemoglobin, diabetes, HRV measurements.
Ivanovic (MS), 2014, Serbia	Clinical Center of Serbia	103/477	60, 71	Retrospective cohort study	ECG	≤ 30 >30	CABG	Age, gender.
Kitahara, 2017, USA	University of Chicago Medicine,	119/486	65/33.7	Retrospective cohort study	NA	<24.9 25–29.9 30–34.9 ≥35	cardiac surgery	Sex, height, weight, dyslipidemia, hypertension, DM, chronic renal failure, renal failure on dialysis, COPD, EF.
Kuduvallia, 2002, UK	Cardiothoracic Centre-Liverpool.	1155/4713	62, 79	Prospectively cohort study	ECG	<30 30–35 >35	CABG	Age, sex, previous cardiac surgery, LVEF, left main stem stenosis, number of major coronary arteries with stenosis >70%, priority of surgery, peripheral vascular disease, DM, renal dysfunction, and respiratory disease.
Lee, 2018, South Korea	Tertiary hospital in Seoul	244/999	65.4, 75.3	Retrospective cohort study	ECG	<25 >25	CABG	Age, Acute coronary syndrome, hypertension, ejection fraction, on pump, Post operation electrolyte Potassium: Potassium, numeral rating scale
Melduni, 2011, USA	Olmsted County, Minnesota	135/351	66.7, 67.2	Prospectivey cohort study	Medical records	Continuous variable	Cardiac Surgery	Age, BMI, hypertension, mitral regurgitation, diastolic function, type of operation, and perfusion time.
Moulton, 1996, USA	Barnes Hospital	833/2299	62.8, 65.1	Retrospective cohort study	NA	≤30 >30	CABG	Age, sex, race, history of reoperation, CHF, prior MI, renal failure, DM, hypertension, COPD or stroke, CPB, aortic cross-clamp.
Omer, 2016, USA	Veterans Affairs hospital	215/1248	62.4, 99	Retrospective cohort study	ECG	<25 25–30 ≥30	CABG	Age, a history of hypertension, obesity, DM, inflammation, and longer pump and cross-clamp times.
Pan, 2006 USA	Texas Heart Institute, St. Luke’s Episcopal Hospital,	1913/9862	62.9, 75.4	Retrospective cohort study	NA	20–24.9 25–29.9 30–34.9 35–39.9 ≥40	CABG	Age, sex, hypertension, pulmonary disease, hyperlipidemia, DM, total bypasstime, *β*-Blocker, antiarrhythmics, EF, triple-vessel CAD, left main CAD, renal insufficiency.
Perrier, 2016, France	University Hospital of Strasbourg	311/1481	65.2, 81.2	Prospectivey cohort study	ECG	≤ 35 >35	CABG	Age, eGFR<60 ml/min, PAD, anti-platelet treatment, CHA2DS2-VASC score, *β*-blockers
Reeves, 2003, UK	Patient Analysis & Tracking System, Dendrite Clinical Systems	675/4372	NA, 81.1	Prospectivey cohort study	ECG	<25 25–30 30–35 ≥35	CABG	age, Parsonnet score, number of grafts, blood loss; red blood cell, platelet, fresh frozen plasma transfusion; postoperative hemoglobin levels; duration of ventilation, ICU stay, combined ICU and HDU stay, and total postoperative stay
Stamou, 2011, USA	Sanger Heart and Vascular Institute	600/2440	62.5, 73.3	Retrospective cohort study	NA	18.5–24.9 25–29.9 ≥30	CABG or valve surgery	Propensity scores.
Stefàno, 2020, Italy	Tertiary hospital in Florence	127/249	65.4, 77.3	Retrospective cohort study	ECG	Continuous variable	CABG or valve surgery	Age, ACEI, statins, operation time, total clamp time, cardiopulmonary bypass time, presence of pericardial/pleural effusion, arterial hypertension, plasmatic creatinine
Sun, 2011, USA	Washington Hospital Center	3462/12367	64.3, 71	Retrospective cohort study	ECG	<18.5 18.5–25 25–30 30–40 ≥40	CABG	Age, sex, Race, HF, Left main coronary artery stenosis, Ventricular arrhythmias, Preop angina, OSA, DM, Hypertension, Family history of CAD, Previous stroke, Hypercholesterolemia, Hemodialysis, Current smoker, *β*-blockers, ACEI, Lipid-lowering drugs.
Tosello, 2015, France	Cardiac Surgery Unit of the Hopital Europeen G	36/176	70.5, 67.6	Prospective cohort study	ECG	Continuous variable	BAVR	Age, sex, weight, heihgt, smoking, DM, CKD, COPD, CAD, PVD, *β*-blocker, amiodarone, LVEF<50%, extracorporeal circulation, aortic cross-clamp time, transfusion in ICU, EUROSCORE >10
Tadic M, 2011, Serbia	Clinical Center of Serbia	72/322	59.9, 71.7	Retrospective cohortstudy	ECG	<30 ≥ 30	CABG	Age, hypertension, DM, obesity, hypercholesterolemia, leukocytosis, and segmental kinetic disturbances of the left ventricle.
Wong, 2015, USA	Stanford University School of Medicine	226/545	66.2, 57.2	Retrospective cohort study	ECG	Continuous variable	CABG, AVR, MVR	Age, sex, previous AF, smoking status, elective status, IABP, COPD, history of cerebral vascular event, surgery
Yap, 2007, Australia	St Vincent’s Hospital and The Gee long Hospital	1425/3968	66.4, 73	Retrospective cohort study	NA	20–30 30–40 ≥40	CABG and valve surgery	Age, sex, DM, hypercholesterolemia, renal impairment (Cr >0.2 mmol/L), preoperative dialysis, hypertension, cere brovascular disease, PVD, COAD, NYHA class IV, severe LV impairment (ejection fraction <30%), mean PA pressure, emergency status and CPB time.
Zacharias, 2005, USA	Saint Vincent Mercy Medical Center and Saint Luke’s Hospital	1496/6749	NA	Retrospective cohort study	ECG, physician findings, hospital or physician chart notes and discharge summaries	<22 22–25 25–30 30–35 35–40 ≥40 Continuous variable	CABG or valve surgery	Age, gender, white race, current smoker, DM, hypertension, PRF, COPD, PVD, MI, CHF, angina , arrhythmia , preoperative medications, triple-vessel disease, LMD, emergency surgery, mitral valve surgery, aortic valve surgery, off-pump, perfusion time, cross-clamp time, and IABP.

**Notes.**

Abbreviation ECG electrocardiograph CABG coronary artery bypass grafting CAD coronary artery disease ACT aortic clamp time BSA body surface area COPD chronic obstructive pulmonary disease PVD peripheral vascular disease MI myocardial infarction LVEF left ventricular ejection fraction ECC extra corporal circulation RBC red blood cell FFP fresh frozen plasma CPB cardiopulmonary bypass EF ejection fraction NYHA New York Heart Association CHF congestive heart failure ITA internal thoracic artery BMI body mass idex DM diabetes mellitus PAD peripheral artery disease STS society of thoracic surgeons PROM predicted risk of operative mortality HRV heart rate variability EF ejection fraction eGFR Estimated Glomerular Filtration Rate ICU intensive care unit HDU high dependency unit HF heart failure ACEI Angiotensin-Converting Enzyme Inhibit CKD chronic kidney disease PVD peripheral vascular disease PA peripheral artery PRF preoperative renal failure LMD left main disease IABP intra-aortic balloon pump DM diabetes mellitus

### Study characteristics and quality

Table 1 shows the detailed characteristics of the included studies. Overall, these studies were published between 1996 and 2020. Among the included studies, the sample sizes ranged from 92 to 18,517 with a total of 141,442 individuals. The mean age varied from 56 to 68 years. Sixteen (17 cohorts) studies were from North America (the US and Canada), Twelven studies were from Europe, one study were from Oceania, and five studies were from Asia. Seventeen studies reported the BMI as a categorical variable, eleven studies reported the BMI as a continuous variable, and two articles reported the BMI as both a categorical and continuous variable.

The overall reporting quality of the included studies was acceptable. All included studies obtained an NOS ≥ 6 points (Table S4).

### Categorical analysis of the effect of BMI on POAF

**Figure 2:**
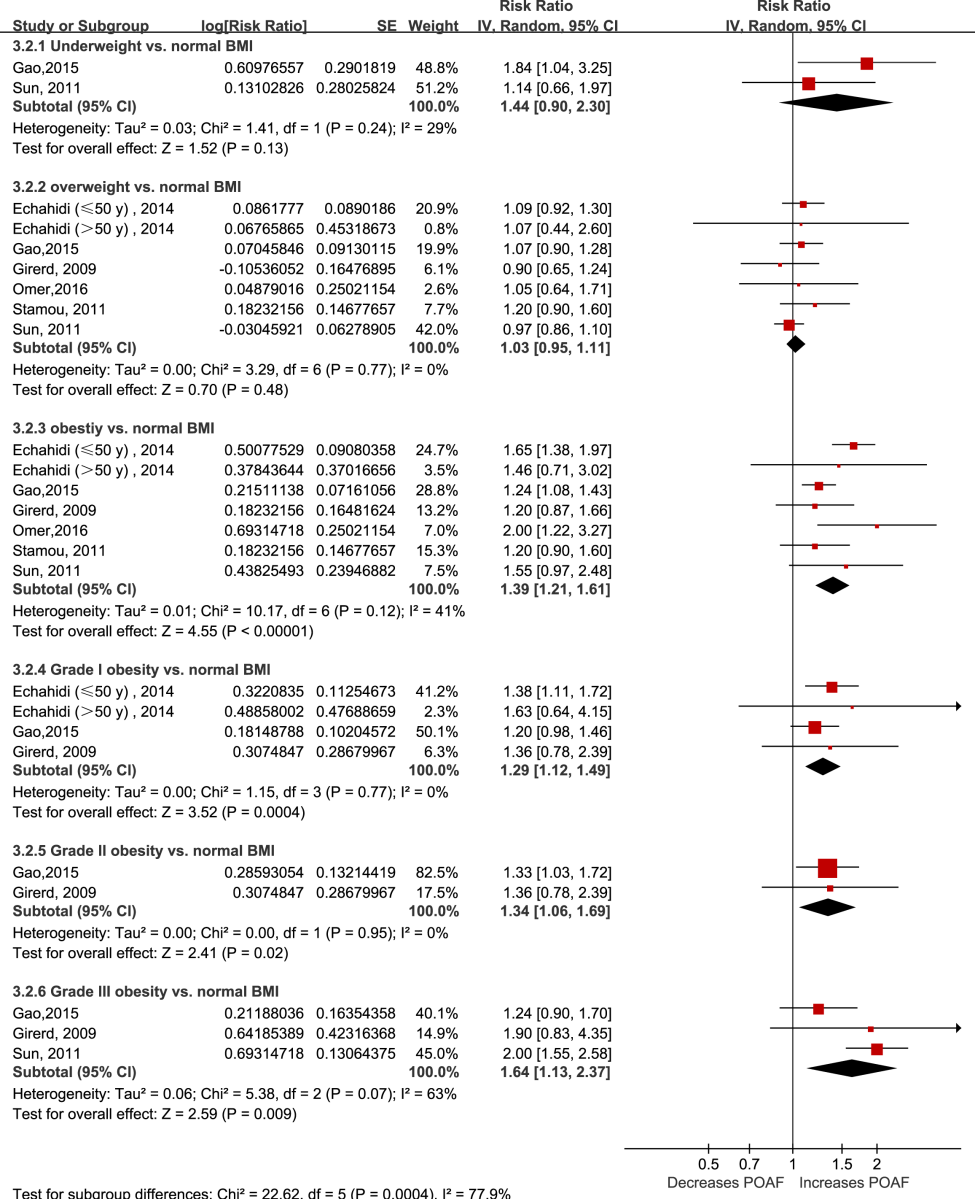
Forest plot of the categorical analysis of the impact of body mass index on POAF. POAF: postoperative atrial fibrillation after cardiac surgery.

Fourteen studies grouped the BMI categories according to the World Health Organization criteria; however, only six articles (seven cohorts) (Echahidi et al., 2007; Gao et al., 2016; Girerd et al., 2009; Omer et al., 2016; Stamou et al., 2011; Sun et al., 2011) reported using normal BMI as the reference group. As shown in Fig. 2, being underweight (RR: 1.44, 95% CI [0.90–2.30]; I^2^ =** 29%; *P* = 0.14) or overweight (RR: 1.03, 95% CI [0.95–1.11]; I^2^ =** 0%; *P* = 0.48) was not associated with an increased risk of POAF. In contrast, obesity significantly increased the risk of POAF (RR: 1.39, 95% CI [1.21–1.61]; I^2^ = 41%; *P* < 0.0001). Interestingly, the risk of POAF seemed to gradually increase with the obesity stage (RR of 1.29 for stage I obesity, 1.34 for stage II obesity, and 1.64 for stage III obesity).

### Dose–response analysis of the effect of BMI on POAF

**Figure 3:**
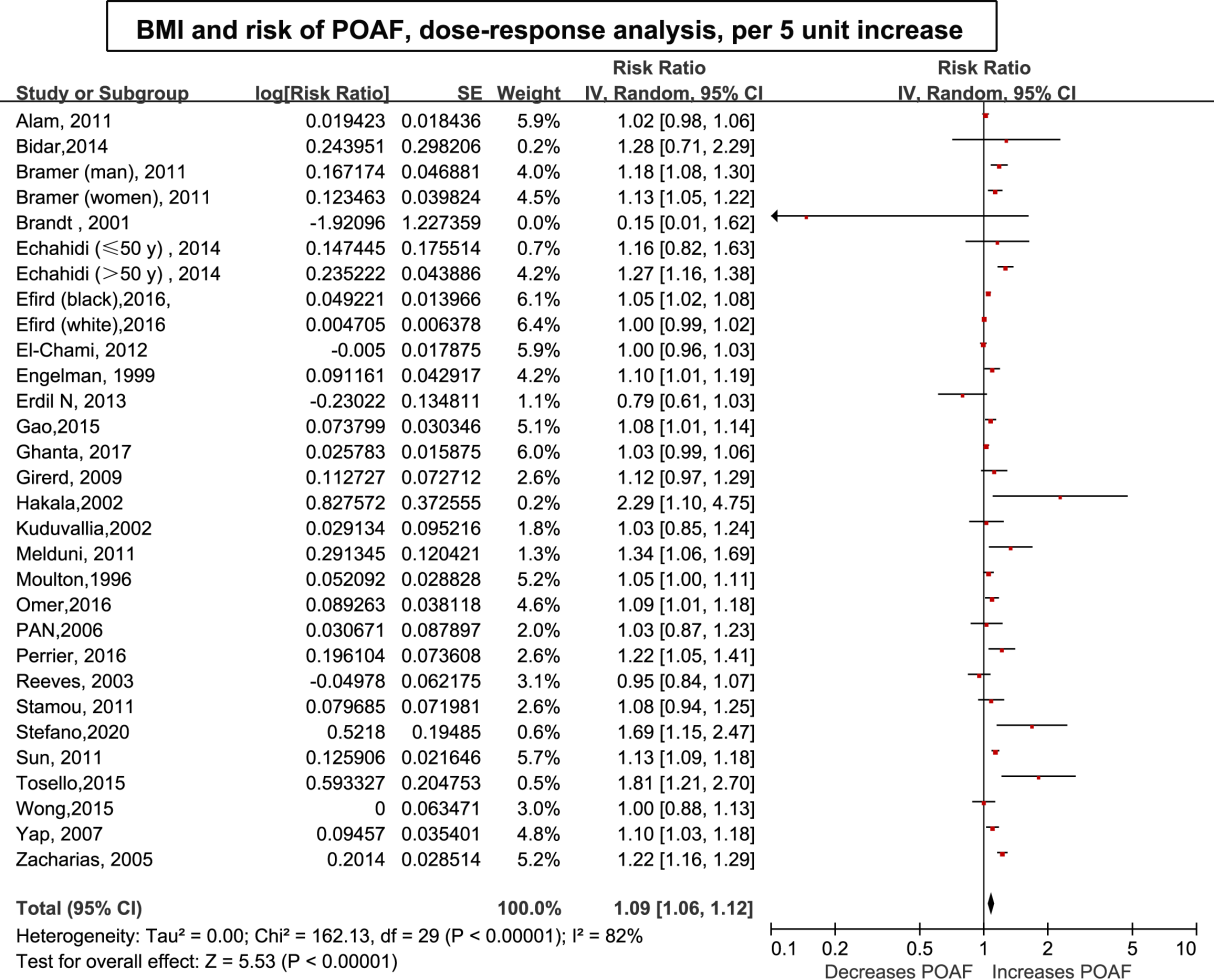
Forest plot of the association between body mass index and POAF and exposure-effect analysis, per five units. POAF: postoperative atrial fibrillation after cardiac surgery.

**Figure 4:**
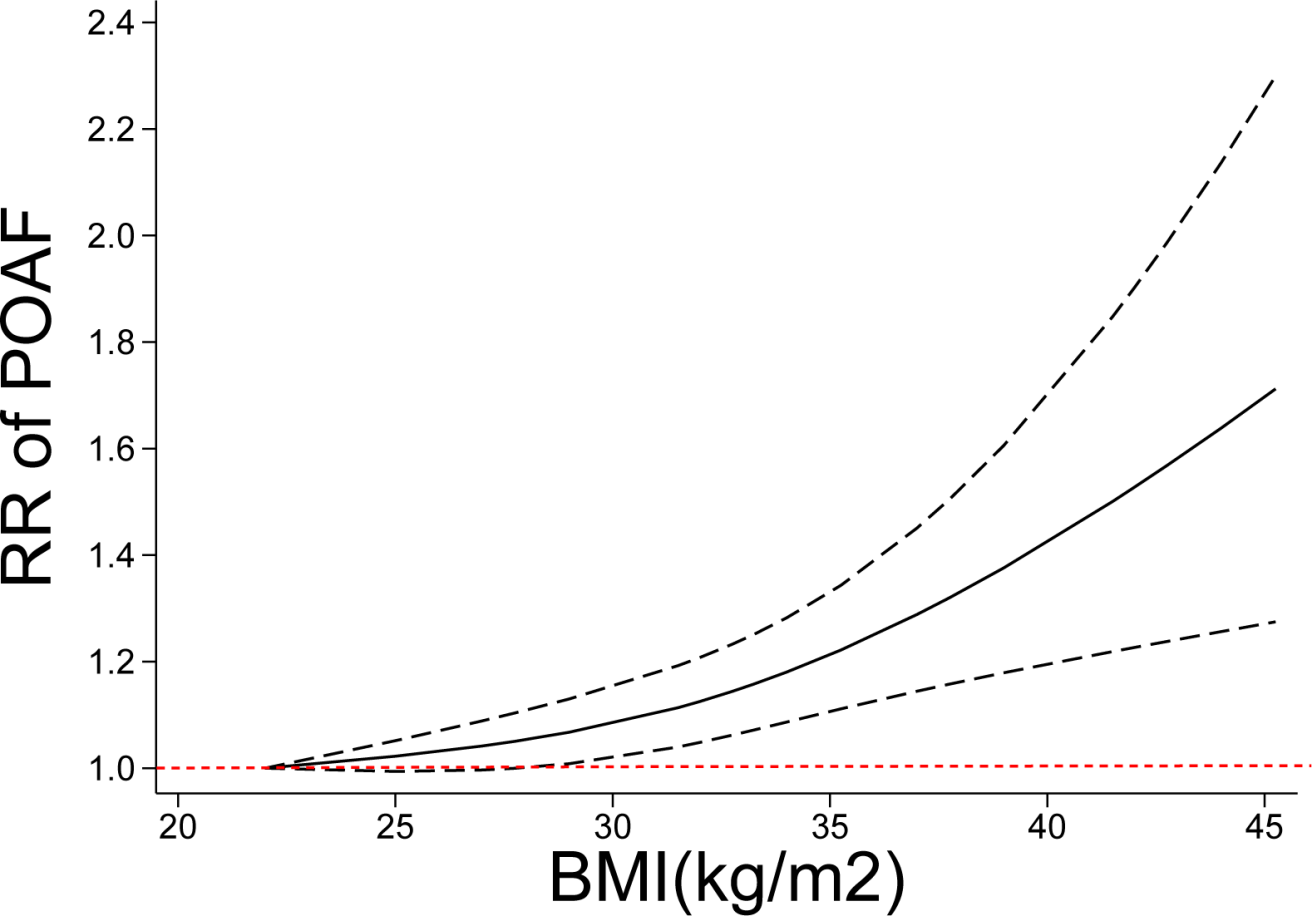
Nonlinear exposure-effect analysis of body mass index and POAF. The solid and dashed lines represent the estimated relative risk and the 95% confidence interval, respectively. POAF: postoperative atrial fibrillation after cardiac surgery.

Thirty-three (34 cohorts) studies (Alam et al., 2011; Banach et al., 2007; Bidar et al., 2014; Bramer et al., 2011; Brandt et al., 2001; Echahidi et al., 2007; Efird et al., 2016; El-Chami et al., 2012; Engelman et al., 1999; Erdil et al., 2014; Gao et al., 2016; Ghanta et al., 2017; Girerd et al., 2009; Gurbuz et al., 2014; Hakala et al., 2009; Ivanovic et al., 2014; Kitahara et al., 2017; Kuduvalli et al., 2002; Lee & Jang, 2020; Melduni et al., 2011; Moulton et al., 1996; Omer et al., 2016; Pan et al., 2006; Perrier et al., 2017; Reeves et al., 2003; Stamou et al., 2011; Stefano et al., 2020; Sun et al., 2011; Tadic, Ivanovic & Zivkovic, 2011; Tosello et al., 2015; Wong et al., 2015b; Yap et al., 2007; Zacharias et al., 2005) involving 33,271 cases/141,442 patients were included in the dose–response analysis of BMI and POAF. The summary RR for a 5-unit increase in BMI was 1.09 (95% CI [1.06–1.12]) with each weight not exceeding 7%. Significant heterogeneity (I^2^ = 82%) (Fig. 3) was found across the studies. In the sensitivity analyses excluding the largest weighted study, the pooled RR ranged from 1.09 (95% CI [1.05–1.12], *P* < 0.001; I2 = 76%) to 1.10 (95% CI [1.06–1.14], *P* < 0.001;I^2^ = 77%). Additionally, the pooled results were not significantly changed when omitting one study at a time (Fig. S1 ). There was no evidence of nonlinearity (*P* = 0.44) in the relationship between BMI and POAF (Fig. 4). The nonlinear curve showed that obesity, but not overweight, significantly increased the risk of POAF compared with the patients with a normal BMI (Fig. 4). Table S5 displays the RR estimates from the nonlinear exposure-effect analysis of selected BMI values; these values were derived from the nonlinear figures.

We further performed a subgroup analysis by type of cardiac operation. Nineteen articles (20 cohorts) reported the association in coronary artery bypass graft (CABG), three articles reported the association in valve surgery, and eleven articles reported the association in combined types of cardiac surgery. The summary RRs for a 5-unit increment in BMI in the CABG group, valve surgery group, and combined cardiac surgery group were 1.07 (95% CI [1.03–1.11], *P* = 0.001; I^2^ = 82%), 1.34 (95% CI [0.81–2.22], *P* = 0.25; I^2^ = 84%), and 1.13 (95% CI [1.06–1.19], *P* < 0.001; I^2^ = 78%), respectively (Table 2). There was still a linear relationship between POAF and BMI in the CABG group (P_nonlinearity_ = 0.12); notably, the risk of POAF significantly increased at a BMI of 30 and rose more steeply at higher BMI levels. However, the curve was somewhat steep in the combined cardiac surgery group **(Fig. S2)**.

**Table 2:** Subgroup analysis of body mass index and post-cardiac operation atrial fibrillation.

**Items**		**Number of studies**	**RR**	**I**^**2**^	***P***	
					**Within subgroup**	**Between subgroup**
Result of primary analysis		30	1.09 [1.05, 1.12]	82	<0.001	NA
Effect model	Random effect	30	1.04 [1.03, 1.04]	82	<0.001	NA
	Fixed effect	30	1.03 [1.03,1.04]	82	<0.001	
Age	≥65	11	1.12 [1.05, 1.20]	78	<0.001	0.15
	<65	15	1.06 [1.02, 1.12]	78	0.002	
Region	Northern America	16	1.07 [1.04, 1.10]	84	<0.001	0.25
	Europe	7	1.23 [1.04, 1.45]	75	0.01	
	Asia	2	0.95 [0.71, 1.27]	70	0.53	
	Oceania	4	1.11[1.06, 1.17]	0	<0.001	
NOS scores	<7 scores	7	1.03 [1.00, 1.05]	69	0.05	<0.001
	≥7 scores	20	1.12 [1.08, 1.16]	64	<0.001	
Publication year	1999-2010	10	1.12 [1.06, 1.17]	58	<0.001	0.25
	2011-2020	20	1.08[1.04, 1.11]	85	<0.001	
AF Diagnosis	ECG	26	1.14 [1.09, 1.19]	67	<0.001	0.33
	Others	4	1.03 [0.97, 1.09]	81	0.37	
Sample size	<1000	8	1.20 [0.93, 1.53]	87	0.16	0.37
	≥ 1000	22	1.07 [1.04, 1.09]	75	<0.001	
Cases	Case <100	4	1.48 [1.11, 1.99]	33	0.008	0.06
	Case ≥100	26	1.08 [1.05, 1.11]	82	<0.001	
Operation type	CABG	17	1.07 [1.03, 1.11]	82	0.001	0.23
	Valve	2	1.34 [0.81, 2.22]	84	0.25	
	Mixed	11	1.13 [1.06, 1.19]	78	<0.001	
Adjusted factors	*Age* (+)	26	1.09 [1.05, 1.12]	85	<0.001	0.64
	*Age* (-)	4	1.33 [0.95, 1.86]	53	<0.001	
	Sex (+)	18	1.07 [1.04, 1.11]	86	<0.001	0.28
	Sex (-)	12	1.12 [1.04, 1.21]	65	0.003	
	DM (+)	17	1.09 [1.05, 1.13]	86	<0.001	0.89
	DM (-)	13	1.08 [1.02, 1.15]	75	0.01	
	Hypertension (+)	14	1.07 [1.04, 1.11]	85	<0.001	0.77
	Hypertension (-)	16	1.09 [1.02, 1.16]	85	<0.001	
	COPD (+)	10	1.13 [1.09, 1.17]	33	<0.001	0.14
	COPD (-)	20	1.08 [1.05, 1.12]	83	<0.001	
	CAD (+)	10	1.09 [1.03, 1.15]	86	<0.001	0.60
	CAD (-)	17	1.07 [1.03, 1.11]	81	<0.001	

**Notes.**

Abbreviation NA not available ECG electrocardiograph CABG coronary artery bypass grafting CAD coronary artery disease COPD chronic obstructive pulmonary disease DM diabetes mellitus

### Waist circumference obesity, visceral adiposity index and POAF

Three studies (Engin et al., 2020; Girerd et al., 2009; Ivanovic et al., 2014) reported an analysis of the association between visceral adiposity and the risk of AF after CABG and included 536 cases among 2,691 participants. Due to the limited number of studies, we did not pool the results. Ivanovic et al. reported that abdominal obesity was associated with an increased risk of new-onset POAF after 72 h, including 545 patients (RR: 1.67) adjusted by age and sex. In another nested case-control study involving 2214 male patients with POAF, a consistent result was found after adjustments (OR: 1.51). (Fig. S3)

In another cohort study, Engin et al. (2020) showed that the visceral adiposity index significantly increased the risk of AF in patients who underwent isolated CABG.

### Subgroup and meta-regression analyses

We conducted a subgroup analysis and a meta-regression by patient characteristics, such as age, region, confounding factors and potential intermediate factors. We found some indication of a stronger relationship between BMI and POAF among the studies with higher NOS scores (Table 2). As shown in Table 2, the positive association between BMI and the risk of POAF persisted in almost all subgroup analyses by age, region, sample size, study quality and adjustment for clinical confounding factors (e.g., age, sex) and intermediates (e.g., COPD, DM), and there was no evidence of heterogeneity among any of these subgroups in the meta-regression analyses.

### Publication bias

A possible lack of publication bias was indicated by Egger’s and Begg’s tests and the funnel plot (Figs. S4–S6).

## Discussion

This study presents the most comprehensive dose–response analysis of the relationship between adiposity and the risk of POAF. By combining 34 cohorts involving 33,271 cases/141,442 patients, we found a 9% increased risk of POAF per a 5-unit increase in BMI. Both the categorical analysis and exposure-effect model showed that obesity, but not being overweight or underweight, significantly increased the risk of POAF. Finally, we also showed abdominal adiposity and the risk of new-onset AF in patients after cardiac surgery. Collectively, these findings provide a comprehensive overview of the association between obesity and POAF. Although the dose–response relationship between obesity and the risk of POAF was reported in a study by Wong et al. (2015a), the shape of the association between BMI and POAF remains unclear, and the associations between overweight and the risk of POAF have not been comprehensively assessed. Several cohort studies (Frost, Hune & Vestergaard, 2005; Wilhelmsen, Rosengren & Lappas, 2001) found that being overweight significantly increased the risk of new onset AF; however, an independent positive relationship in POAF was not confirmed in several large studies with long-term follow-up after adjusting for clinical confounding variables (Murphy et al., 2006; Wang et al., 2004) (e.g., the Framingham Heart Study). Consistently, our categorical analysis and exposure-effect analysis uniformly showed that being overweight did not statically increase the risk of POAF. This result was unsurprising. First, as described by a previously published meta-analysis (Hernandez et al., 2013; Phan et al., 2016), the magnitude of the association between overall obesity and POAF is minimal, with an RR of 1.12−1.21. Consistently, our results also showed that the summary RR per 5 units of BMI was 9%, further suggesting that the real effect may even be very small in magnitude. Furthermore, the risk factors for POAF are complicated and mainly include transient perioperative factors and a pre-existing condition (Dobrev et al., 2019). A large cohort study (Lubitz et al., 2015) found that the repeat recurrence of AF in cardiac surgery was higher than that in noncardiac operations, supporting that the important role of transient factors (e.g., cross-clamp time and intra-aortic balloon pump) in contributing POAF. Thus, we speculated that the pathological condition caused by overweight might be compensable and insufficient to trigger POAF independently.

The association between obesity and atrial fibrillation is not new in the field of cardiac surgery. However, due to the limitations of univariate analyses and lack of evaluation of other clinical factors or potential intermediates in previously published meta-analyses (Hernandez et al., 2013; Phan et al., 2016), the multitude of other factors and potential intermediates (e.g., age, smoking, obstructive sleep apnea (OSA), COPD, and hypertension) that may also affect the association between obesity and POAF incidence are still unclear. For example, the association of hypertension with POAF was slightly stronger than that of obesity and was more common in cardiac surgery patients with obesity. Stamou et al. (2011) found that 87% of patients with obesity had preoperative hypertension compared to 75% of patients without obesity in a cohort of 2,465 patients undergoing cardiac surgery. Another important intermediate factor is lung disease. For example, a study reported that the incidence of POAF increases with surgical invasiveness from an RR of 2.26 after mediastinal surgery to 8.90 in patients undergoing pneumonectomy, suggesting that a strong association exists between lung disease and POAF (Vaporciyan et al., 2004). More consistently, several studies also showed that COPD was strongly linked to the incidence and progression of AF (Durheim et al., 2018; Grymonprez et al., 2019). Another study also showed that POAF was associated primarily with metabolic syndrome (OR, 2.36; *p* = 0.02) rather than BMI in a younger population (Echahidi et al., 2007). Our results showed that the positive association persisted in almost all subgroup analyses by age, region, number of cases, and study quality and after adjustment for these abovementioned factors, indicating an independent association between obesity and the risk of POAF.

Notably, not all important clinical confounding factors or potential intermediates were assessed in the present study. For example, OSA was common in patients with obesity and sharply increased with BMI. The link between OSA and new onset incident AF has been confirmed (Cadby et al., 2015). Various studies have found that OSA is independently associated with an increased risk of AF following CABG (Qaddoura et al., 2014; van Oosten et al., 2014). Thus, some authors have suggested that the positive correlation between obesity and POAF can be explained by OSA. Similarly, LAD enlargement, which is another pathological condition that often coexisted with obesity, was also identified as a crucial factor that independently contributes to the incidence of AF (Tsang et al., 2001). However, few included studies adjusted for OSA or enlarged LAD, and even fewer studies examined the direct link among OSA, LAD and increased POAF. Therefore, the role of OSA and LAD in the relationship between BMI and POAF is still unclear and needs to be further studied.

Notably, BMI is commonly used because it is simple to apply and inexpensive. However, the utility of BMI in evaluating obesity has been criticized because it is indistinguishable from fat. In our results, body fat measured by waist circumference increased the risk of POAF by 51%–67%. This value was much greater than that previously reported in the association between obesity and POAF and the value observed in our study. Although the included study was limited, some authors have highlighted the importance of using multiple measures, such as visceral obesity, in the risk assessment of POAF (Girerd et al., 2009).

The effect of POAF on secondary outcomes was not assessed in the present study. Previous studies have observed an “obesity paradox” in the outcomes of patients undergoing cardiac surgery, with individuals affected by overweight and class I and II obesity having lower mortality (Mariscalco et al., 2017; Stamou et al., 2011). However, recent studies using dose–response methods and more comprehensive meta-analyses did not find any protective effect or higher mortality among patients with extreme obesity (Liu et al., 2020). Consistently, another meta-analysis found higher major morbidity and total hospital costs in patients with obesity undergoing cardiac surgery (Ghanta et al., 2017). Our results further support this observation of a lack of an obesity paradox; we found that POAF increased with increasing BMI, and morbidly increased POAF by 40% and 120% in the total cardiac surgery and CABG subgroups, respectively. POAF independently predicted stroke (Lin et al., 2019) and long-term mortality. Thus, regarding obesity, especially morbid obesity, the risk of POAF should be carefully evaluated before cardiac surgery, and specific interventions for the prevention of POAF should be considered. To date, purposeful weight loss has been shown to reverse many changes in cardiac performance and morphology associated with obesity and the incidence and burden of AF (Lavie et al., 2017; Middeldorp et al., 2018). However, weight loss might not be as prevalent in the cardiac surgery subgroup because most CABG procedures are emergent and unexpected or conducted in patients with poor cardiac function (patients receiving valve replace). Alternatively, the administration of certain medications was the most commonly used therapy for POAF prophylaxis. *β*-blockers were uniformly recommended by the guidelines (class I recommendation) (Andrade et al., 2018; Fuster, 2006; Kirchhof et al., 2016). A combination of *β*-blockers and amiodarone also can be considered for POAF prophylaxis in these high-risk patients.

Finally, our findings have important clinical implications for the prevention of POAF. Since previous meta-analysis analyzed BMI but did not include other measures of fat in relation to the risk of POAF, and did not assess the dose–response relationship between BMI and POAF in as great detail as the present analysis. In addition, our results show that obesity was associated with an increased risk of POAF in studies from Europe, North America, and Oceania, suggesting that the prevention of obesity is essential across populations. The current analysis suggests that both general and abdominal adiposity (waist circumference) measures are related to an increased risk of POAF and that being relatively slim, as assessed by BMI and other adiposity measures, may confer the lowest risk of POAF.

### Strengths and limitations

Our meta-analysis has several strengths. First, all included studies were designed as cohort studies, largely reducing the possibility of selection bias. Second, this meta-analysis included a large number of cohort studies (33 studies) with BMI reported as either a categorical or continuous variable, providing strong statistical power to detect moderate associations. The detailed exposure–effect analyses clarified the shape of the exposure–effect relationship. Third, the positive association between BMI and POAF persisted in different subgroups (e.g., age or region) and after adjusting for confounding factors, confirming the robustness of our findings.

Our study inevitably has several limitations. First, this meta-analysis included observational studies, and bias was not entirely avoided. Measured and unmeasured confounding variables might have influenced our results. However, this limitation cannot be mitigated by the large number of studies and adjustments for coexistent confounding factors in all included studies. Second, significant heterogeneity (I^2^ =82%) was observed across the included studies, which might have been derived from between-study differences, such as differences in study design, basic patient characteristics, and analytical strategies. Third, we did not analyze the long-term POAF incidence since most studies only reported the incidence of POAF during the length of hospitalization. However, most POAF has a peak incidence between days 2 and 4 after surgery (Dobrev et al., 2019), especially during the first postoperative week. Fourth, we did not assess the effect of BMI on secondary outcomes in patients undergoing a cardiac operation, such as stroke and death, which have been thoroughly studied in previous studies (Ghanta et al., 2017; Liu et al., 2020).

## Conclusion

Based on the current evidence, Our results showed that high body mass index or abdominal adiposity was independently associated with an increased risk of POAF, while being underweight or overweight might not significantly increase the POAF risk.

##  Supplemental Information

10.7717/peerj.11855/supp-1 Supplemental Information 1 Prisma checklist

10.7717/peerj.11855/supp-2 Supplemental Information 2 Rationale and contribution

10.7717/peerj.11855/supp-3 Supplemental Information 3 Supplemental Materials

## Additional Information and Declarations

### Data Availability

The following information was supplied regarding data availability: All data generated or analyzed during this study are in the Supplemental Files.
