# Supplementary material for: Dose-response relationship among body mass index, abdominal adiposity and atrial fibrillation in patients undergoing cardiac surgery: a meta-analysis of 35 cohorts
Source: PeerJ. 2021 Jul 21;9:e11855. doi: 10.7717/peerj.11855 (PMC8308618; doi:10.7717/peerj.11855)
Supplement: Supplemental Information 2 [file peerj-09-11855-s002.docx]

**Statement of Rationale and Contribution of the work.**

**1.The rationale for conducting the meta-analysis**

Postoperative atrial fibrillation (POAF) is among the most common complications arising from cardiac surgery, affecting between 20% and 50% of patients, and is associated with significantly worse adverse outcomes (such as all-cause death and stroke). However, the no comprehensive study has quantitatively assessed the exposure- effect relationship between body mass index and POAF. The shape of the exposure–effect curve and whether being overweight increases the risk of POAF are still unclear. Moreover, no multi-univariate meta-analysis had explored the clinical factor(such as chronic obstructive pulmonary disease (COPD), smoking, diabetes, and left atrial diameter (LAD)) in the association between body mass index and POAF.

**2. The contribution that the meta-analysis makes to knowledge in light of previously published related reports, including other meta-analyses and systematic reviews.**

1.Being underweight or overweight might not significantly increase the POAF risk.

2.Abdominal obesity was associated with an increased risk of POAF.

3.There is a linear positive association between body mass index and POAF
