# Supplementary material for: Dose-response relationship among body mass index, abdominal adiposity and atrial fibrillation in patients undergoing cardiac surgery: a meta-analysis of 35 cohorts
Source: PeerJ. 2021 Jul 21;9:e11855. doi: 10.7717/peerj.11855 (PMC8308618; doi:10.7717/peerj.11855)
Supplement: Supplemental Information 3 [file peerj-09-11855-s003.docx]

**Dose-response relationship among body mass index, abdominal adiposity and atrial fibrillation in patients undergoing cardiac surgery: a meta-analysis of 35 cohorts**

**SUPPLEMENTAL METHODS**

**Literature Search**

We systematically searched the Cochrane Library, PubMed, and Embase databases for eligible studies from inception through April 1, 2021. Three groups of keywords or MeSH terms (linked to body mass index, waist circumference, and waist-to-hip ratio, adiposity, abdominal adiposity or visceral adiposity, atrial fibrillation and cardiac surgery) were combined using the Boolean operator "and". In addition, we searched the reference lists of other relevant publications to identify further studies. No language restrictions were applied in the whole literature search.

**Study Selection**

Studies were considered eligible if they: (1) designed as prospective studies including post-hoc analysis of randomized controlled trials (RCTs) or observational studies(cohort or nest case-control); (2) reported the adjusted RR for association btewwen body mass index (BMI), waist circumference, and waist-to-hip ratio on atrial fibrillation after cardiac surgery(POAF); (3) made available a quantitative measure of adiposity and the number of POAF cases in each adiposity category for the dose-response analysis. For multiple publications/reports created from the same data, the studies with the l the largest number of POAF cases were included. In addition, certain publication types (e.g., reviews, editorials, letters, conference abstracts, and animal studies), or studies with insufficient data were excluded from this analysis.

**Data Extraction and Quality Assessment**

For each study, the basic characteristics were extracted, mainly including the first author, publication year, geographical location, study type, participants (sex, age, and sample size), duration of follow-up, adjustments for confounders, categories of adiposity and adjusted risk ratios (RRs) with its 95% confidence intervals (CIs) for each adiposity category. If both unadjusted and adjusted RRs existed in one study, we extracted the most completely adjusted one.

Post-hoc analyses of RCTs can be equivalent to observational studies.^1^ Therefore, we used the Newcastle-Ottawa quality assessment scale (NOS) to evaluate the quality for all included studies.^2^ The validated NOS items with a total of 9 stars involved three aspects including the selection of cohorts, the comparability of cohorts, and the assessment of the outcome.^3^ In this meta-analysis, a NOS score of ≥6 stars was regarded as moderate- to high-quality, otherwise, as low-quality studies.^4^

**Statistical Analyses**

Summary RRs and 95% CIs for a 5 unit increment in BMI were using a random effects model. We calculated study-specific slopes (linear trends) and 95% CIs from the natural logs of the reported RRs and CIs across categories of adiposity measures adiposity by using the method of Greenland and Longnecker^5^. We performed the non-linear dose-response analysis by using the robust error meta-regression method described by *Xu et al*.^6^ This method is based on a “one-stage approach” which treating each study as a cluster of the whole sample and considering the within study correlations by clustered robust error. It requires known levels of adiposity and RRs with variance estimates for at least two quantitative exposure categories.^6^ For studies that did not set the lowest adiposity group as a reference, data were transformed using a method described by *Hamling et al*.^7^ which requires the number of cases and participants in each category. If these data could not be obtained from an article, the evidence was not pooled. If the median or mean adiposity was not provided and reported in ranges, we estimated the midpoint of each category by averaging the lower and upper boundaries of that category. If the highest or lowest category was open-ended, we assumed that the open-ended interval length was the same as the adjacent interval.^8^ To assess the heterogeneity of RRs across studies, the I*^2^* (95% CI) statistic was calculated with the following interpretation: low heterogeneity, defined as I*^2^* < 50%; moderate heterogeneity, defined as I*^2^* 50% to 75%; and high heterogeneity, defined as I*^2^* >75%.^9^ Moreover, subgroup analyses were not carried out when the numbers of included studies in outcomes were limited (≤5). A *P* value < 0.05 was considered statistically significant.

**References:**

1 Gu WJ, Wang F, Tang L, Liu JC. Single-dose etomidate does not increase mortality in patients with sepsis: a systematic review and meta-analysis of randomized controlled trials and observational studies. *CHEST*. 2015; 147: 335-46.

2 Aune D, Greenwood DC, Chan DSM*, et al*. Body mass index, abdominal fatness and pancreatic cancer risk: a systematic review and non-linear dose-response meta-analysis of prospective studies. *Annals of oncology : official journal of the European Society for Medical Oncology / ESMO*. 2012; 23: 843-52.

3 Stang A. Critical evaluation of the Newcastle-Ottawa scale for the assessment of the quality of nonrandomized studies in meta-analyses. *EUR J EPIDEMIOL*. 2010; 25: 603-05.

4 Zhu W, Wan R, Liu F*, et al*. Relation of Body Mass Index With Adverse Outcomes Among Patients With Atrial Fibrillation: A Meta‐Analysis and Systematic Review. *J AM HEART ASSOC*. 2016; 5: e4006.

5 Greenland S. Quantitative methods in the review of epidemiologic literature. *EPIDEMIOL REV*. 1987; 9: 1-30.

6 Doi SAR, Sar D. The robust error meta-regression method for dose-response meta-analysis..

7 Hamling J, Lee P, Weitkunat R, Ambuhl M. Facilitating meta-analyses by deriving relative effect and precision estimates for alternative comparisons from a set of estimates presented by exposure level or disease category. *STAT MED*. 2008; 27: 954-70.

8 Liu T, Xu C, Rota M*, et al*. Sleep duration and risk of all-cause mortality: A flexible, non-linear, meta-regression of 40 prospective cohort studies. *SLEEP MED REV*. 2017; 32: 28-36.

9 Zhu W, Fu L, Ying D*, et al*. Meta-analysis of ATRIA versus CHA 2 DS 2 -VASc for predicting stroke and thromboembolism in patients with atrial fibrillation. *INT J CARDIOL*. 2017; 227: 436-42.

**SUPPLEMENTAL TABLES**

**Table S1: Search strategy**

| Search Terms | Search Options |
| --- | --- |
| #1 | Body mass index |
| #2 | Obesity |
| #3 | Overweight |
| #4 | waist circumference |
| #5 | waist-to-hip ratio |
| #6 | Fat |
| #7 | adiposity |
| #8 | abdominal adiposity |
| #9 | visceral adiposity |
| #10 | Atrial fibrillation |
| #11 | Arrhythmia |
| #12 | Cardiac surgery |
| #13 | #1 OR #2 OR #3 OR #4 OR #5 OR #6 OR #7 OR #8 OR #9 |
| #14 | #10 OR #11 |
| #15 | #13 AND #14 AND #12 |
|  |  |

**Table S2:** PRISMA CHECKLIST

| **Section/topic** | **#** | **Checklist item** | **Reported on page #** |
| --- | --- | --- | --- |
| **TITLE** | | |  |
| Title | 1 | Identify the report as a systematic review, meta-analysis, or both. | 1 |
| **ABSTRACT** | | |  |
| Structured summary | 2 | Provide a structured summary including, as applicable: background; objectives; data sources; study eligibility criteria, participants, and interventions; study appraisal and synthesis methods; results; limitations; conclusions and implications of key findings; systematic review registration number. | 1-2 |
| **INTRODUCTION** | | |  |
| Rationale | 3 | Describe the rationale for the review in the context of what is already known. | 3 |
| Objectives | 4 | Provide an explicit statement of questions being addressed with reference to participants, interventions, comparisons, outcomes, and study design (PICOS). | 3-4 |
| **METHODS** | | |  |
| Protocol and registration | 5 | Indicate if a review protocol exists, if and where it can be accessed (e.g., Web address), and, if available, provide registration information including registration number. | 4 |
| Eligibility criteria | 6 | Specify study characteristics (e.g., PICOS, length of follow-up) and report characteristics (e.g., years considered, language, publication status) used as criteria for eligibility, giving rationale. | 4 |
| Information sources | 7 | Describe all information sources (e.g., databases with dates of coverage, contact with study authors to identify additional studies) in the search and date last searched. | 4 |
| Search | 8 | Present full electronic search strategy for at least one database, including any limits used, such that it could be repeated. | 4 |
| Study selection | 9 | State the process for selecting studies (i.e., screening, eligibility, included in systematic review, and, if applicable, included in the meta-analysis). | 4 |
| Data collection process | 10 | Describe method of data extraction from reports (e.g., piloted forms, independently, in duplicate) and any processes for obtaining and confirming data from investigators. | 4 |
| Data items | 11 | List and define all variables for which data were sought (e.g., PICOS, funding sources) and any assumptions and simplifications made. | 4-5 |
| Risk of bias in individual studies | 12 | Describe methods used for assessing risk of bias of individual studies (including specification of whether this was done at the study or outcome level), and how this information is to be used in any data synthesis. | 4-5 |
| Summary measures | 13 | State the principal summary measures (e.g., risk ratio, difference in means). | 5 |
| Synthesis of results | 14 | Describe the methods of handling data and combining results of studies, if done, including measures of consistency (e.g., I^2^) for each meta-analysis. | 5 |

Page 1 of 2

| **Section/topic** | **#** | **Checklist item** | **Reported on page #** |
| --- | --- | --- | --- |
| Risk of bias across studies | 15 | Specify any assessment of risk of bias that may affect the cumulative evidence (e.g., publication bias, selective reporting within studies). | 5 |
| Additional analyses | 16 | Describe methods of additional analyses (e.g., sensitivity or subgroup analyses, meta-regression), if done, indicating which were pre-specified. | 5 |
| **RESULTS** | | |  |
| Study selection | 17 | Give numbers of studies screened, assessed for eligibility, and included in the review, with reasons for exclusions at each stage, ideally with a flow diagram. | 5 |
| Study characteristics | 18 | For each study, present characteristics for which data were extracted (e.g., study size, PICOS, follow-up period) and provide the citations. | 5-6 |
| Risk of bias within studies | 19 | Present data on risk of bias of each study and, if available, any outcome level assessment (see item 12). | 6 |
| Results of individual studies | 20 | For all outcomes considered (benefits or harms), present, for each study: (a) simple summary data for each intervention group (b) effect estimates and confidence intervals, ideally with a forest plot. | 6 |
| Synthesis of results | 21 | Present results of each meta-analysis done, including confidence intervals and measures of consistency. | 6-7 |
| Risk of bias across studies | 22 | Present results of any assessment of risk of bias across studies (see Item 15). | 7 |
| Additional analysis | 23 | Give results of additional analyses, if done (e.g., sensitivity or subgroup analyses, meta-regression [see Item 16]). | 7 |
| **DISCUSSION** | | |  |
| Summary of evidence | 24 | Summarize the main findings including the strength of evidence for each main outcome; consider their relevance to key groups (e.g., healthcare providers, users, and policy makers). | 7 |
| Limitations | 25 | Discuss limitations at study and outcome level (e.g., risk of bias), and at review-level (e.g., incomplete retrieval of identified research, reporting bias). | 8-9 |
| Conclusions | 26 | Provide a general interpretation of the results in the context of other evidence, and implications for future research. | 10 |
| **FUNDING** | | |  |
| Funding | 27 | Describe sources of funding for the systematic review and other support (e.g., supply of data); role of funders for the systematic review. | 12 |

*From:*  Moher D, Liberati A, Tetzlaff J, Altman DG, The PRISMA Group (2009). Preferred Reporting Items for Systematic Reviews and Meta-Analyses: The PRISMA Statement. PLoS Med 6(6): e1000097. doi:10.1371/journal.pmed1000097

For more information, visit: **www.prisma-statement.org**.

Page 2 of 2

**Table S3. Studies excluded (n=25) with reasons**

| **Studies not included** | **Reasons (According to PICOS)** |
| --- | --- |
| Phan, 2016[1] | Without target data set:This is a meta-analysis |
| Auer, 2005[2] | Not the target exposure: BMI information was insufficient. |
| Chua, 2015[3] | Not the target exposure: BMI information was insufficient. |
| Dandale, 2014[4] | Without target data set: Only providing RR without 95%CI. |
| Drossos, 2014[5] | Not the target exposure: Pericardial fat. |
| Kokkonen, 2005[6] | Not the target exposure: Postoperative hemodynamics and low postoperative serum triiodothyronine. |
| Massom, 2014[7] | Not the target exposure: Circulating cardiac biomarkers (NT-proBNP and hs-cTnT). |
| Mauermann, 2010[8] | Not the target exposure: Hemofiltration during cardiopulmonary bypass. |
| Nishi, 2012[9] | Not the target exposure: Only mentioned in the baseline characteristics of study patients. |
| O’Neal, 2013[10] | Not the target exposure and outcome: Study the relationship between postoperative atrial fibrillation and long-term survival. |
| Parsaee, 2014[11] | Not the target exposure: BMI information was insufficient. |
| Pillarisetti, 2014[12] | Not the target exposure and outcome: Study the relationship between early postoperative atrial fibrillation and late recurrence of AF. |
| Shirzad M, 2010[13] | Not the target exposure: BMI information was insufficient. |
| Weidinger, 2014[14] | Not the target exposure: BMI categories was insufficient. |
| B. Richter, 2006[15] | Not the target population: Patients referred for catheter ablation of AF. |
| Jidéus, 2000[16] | Without target data set: Not providing BMI categories. |
| Ducceschi, 1999[17] | Not the target exposure: BMI information was insufficient. |
| Ahlsson, 2009[18] | Not the target exposure: BMI information was insufficient. |
| Hravnak, 2002[19] | Not the target exposure: BMI information was insufficient. |
| Gürbüz, 2014[20] | Count data or univariate analysis |
| Cao, 2013[21] | Count data or univariate analysis |
| Kinoshita, 2011[22] | Count data or univariate analysis |
| Lopez-Delgado, 2015 | Count data or univariate analysis |
| Orhan, 2004[23] | Count data or univariate analysis |
| Wigfield, 2006[24] | Count data or univariate analysis |
| Yin, 2015[25] | Count data or univariate analysis |

[1] Phan K, Khuong JN, Xu J, Kanagaratnam A, Yan TD. Obesity and postoperative atrial fibrillation in patients undergoing cardiac surgery: Systematic review and meta-analysis. International Journal of Cardiology. 2016;217:49-57.

[2] Auer J, Weber T, Berent R, Ng CK, Lamm G, Eber B. Risk factors of postoperative atrial fibrillation after cardiac surgery. J Card Surg. 2005;20:425-31.

[3] Chua SK, Shyu KG, Lu MJ, Hung HF, Cheng JJ, Chiu CZ, et al. Renal dysfunction and the risk of postoperative atrial fibrillation after cardiac surgery: role beyond the CHA2DS2-VASc score. Europace. 2015;17:1363-70.

[4] Dandale R, Rossi A, Onorati F, Krapivsky A, Kekes P, Milano A, et al. Does aortic valve disease etiology predict postoperative atrial fibrillation in patients undergoing aortic valve surgery? Future Cardiol. 2014;10:707-15.

[5] Drossos G, Koutsogiannidis CP, Ananiadou O, Kapsas G, Ampatzidou F, Madesis A, et al. Pericardial fat is strongly associated with atrial fibrillation after coronary artery bypass graft surgerydagger. Eur J Cardiothorac Surg. 2014;46:1014-20; discussion 20.

[6] Kokkonen L, Majahalme S, Kööbi T, Virtanen V, Salmi J, Huhtala H, et al. Atrial fibrillation in elderly patients after cardiac surgery: Postoperative hemodynamics and low postoperative serum triiodothyronine. Journal of Cardiothoracic and Vascular Anesthesia. 2005;19:182-7.

[7] Masson S, Wu JH, Simon C, Barlera S, Marchioli R, Mariani J, et al. Circulating cardiac biomarkers and postoperative atrial fibrillation in the OPERA trial. Eur J Clin Invest. 2015;45:170-8.

[8] Mauermann WJ, Nuttall GA, Cook DJ, Hanson AC, Schroeder DR, Oliver WC. Hemofiltration during cardiopulmonary bypass does not decrease the incidence of atrial fibrillation after cardiac surgery. Anesth Analg. 2010;110:329-34.

[9] Nishi H, Sakaguchi T, Miyagawa S, Yoshikawa Y, Fukushima S, Saito S, et al. Atrial fibrillation occurring early after cardiovascular surgery: impact of the surgical procedure. Surgery Today. 2012;43:1134-9.

[10] O'Neal WT, Efird JT, Davies SW, O'Neal JB, Anderson CA, Ferguson TB, et al. Impact of race and postoperative atrial fibrillation on long-term survival after coronary artery bypass grafting. J Card Surg. 2013;28:484-91.

[11] Parsaee M, Moradi B, Esmaeilzadeh M, Haghjoo M, Bakhshandeh H, Sari L. New onset atrial fibrillation after coronary artery bypasses grafting; an evaluation of mechanical left atrial function. Arch Iran Med. 2014;17:501-6.

[12] Pillarisetti J, Patel A, Bommana S, Guda R, Falbe J, Zorn GT, et al. Atrial fibrillation following open heart surgery: long-term incidence and prognosis. J Interv Card Electrophysiol. 2014;39:69-75.

[13] Shirzad M, Karimi A, Tazik M, Aramin H, Ahmadi SH, Davoodi S, et al. Determinants of Postoperative Atrial Fibrillation and Associated Resource Utilization in Cardiac Surgery. Revista Española de Cardiología (English Edition). 2010;63:1054-60.

[14] Weidinger F, Schachner T, Bonaros N, Hofauer B, Lehr EJ, Vesely M, et al. Predictors and consequences of postoperative atrial fibrillation following robotic totally endoscopic coronary bypass surgery. Eur J Cardiothorac Surg. 2014;45:318-22.

[15] Richter B, Gwechenberger M, Filzmoser P, Marx M, Lercher P, Gossinger HD. Is inducibility of atrial fibrillation after radio frequency ablation really a relevant prognostic factor? Eur Heart J. 2006;27:2553-9.

[16] Jidéus L, Blomström P, Nilsson L, Stridsberg M, Hansell P, Blomström-Lundqvist C. Tachyarrhythmias and triggering factors for atrial fibrillation after coronary artery bypass operations. Annals of Thoracic Surgery. 2000;69:1064-9.

[17] Ducceschi. Perioperative clinical predictors of atrial fibrillation occurrence following coronary artery surgery. European Journal of Cardio-Thoracic Surgery. 1999;16:435-9.

[18] Ahlsson A, Bodin L, Fengsrud E, Englund A. Patients with postoperative atrial fibrillation have a doubled cardiovascular mortality. Scandinavian Cardiovascular Journal Scj. 2009;43:330.

[19] Hravnak M, Hoffman LA, Saul MI, Zullo TG, Whitman GR, Griffith BP. Predictors and impact of atrial fibrillation after isolated coronary artery bypass grafting. Critical Care Medicine. 2002;30:330.

[20] Gurbuz HA, Durukan AB, Salman N, Ucar HI, Yorgancioglu C. Obesity is still a risk factor in coronary artery bypass surgery. Anadolu Kardiyol Derg. 2014;14:631-7.

[21] Cao H, Zhou Q, Wu Y, Li Q, Roe OD, Chen Y, et al. Preoperative serum soluble receptor activator of nuclear factor-kappaB ligand and osteoprotegerin predict postoperative atrial fibrillation in patients undergoing cardiac valve surgery. Ann Thorac Surg. 2013;96:800-6.

[22] Kinoshita T, Asai T, Suzuki T, Kambara A, Matsubayashi K. Preoperative hemoglobin A1c predicts atrial fibrillation after off-pump coronary bypass surgery. Eur J Cardiothorac Surg. 2012;41:102-7.

[23] Orhan G, Bicer Y, Aka SA, Sargin M, Simsek S, Senay S, et al. Coronary artery bypass graft operations can be performed safely in obese patients. Eur J Cardiothorac Surg. 2004;25:212-7.

[24] Wigfield CH, Lindsey JD, Munoz A, Chopra PS, Edwards NM, Love RB. Is extreme obesity a risk factor for cardiac surgery? An analysis of patients with a BMI > or = 40. Eur J Cardiothorac Surg. 2006;29:434-40.

[25] Yin L, Ling X, Zhang Y, Shen H, Min J, Xi W, et al. CHADS2 and CHA2DS2-VASc scoring systems for predicting atrial fibrillation following cardiac valve surgery. PLoS One. 2015;10:e0123858.

**Table S4**. Quality assessment of included studies

| Author  (Publication Year) | Newcastle-Ottawa Scale | | | | | | | | | |  |
| --- | --- | --- | --- | --- | --- | --- | --- | --- | --- | --- | --- |
|  | Selection | | | Comparability | | | Outcome | | | Total | Average  score |
|  | a | b | c | d | e | f | g | h | i |  |  |
| Alam, 2011 | * | * | * |  | * | * | * |  |  | 6 | 7.6 |
| Bramer, 2011 | * | * | * | * | * | * | * | * | * | 9 |  |
| Brandt, 2001 | * | * | * |  | * | * | * | * | * | 8 |  |
| Banach, 2007 | * | * | * |  | * | * | * | * | * | 8 |  |
| Bidar, 2014 | * | * | * |  | * | * |  | * |  | 6 |  |
| Engelman, 1999 | * | * | * | * | * | * |  |  |  | 6 |  |
| El-Chami, 2012 | * | * | * | * | * | * |  |  |  | 6 |  |
| Erbil N, 2013 | * | * | * | * | * | * | * | * | * | 9 |  |
| Efird, 2016 | * | * | * |  | * | * |  |  |  | 6 |  |
| Echahidi, 2014 | * | * | * | * | * | * | * | * | * | 9 |  |
| Gao, 2016 | * | * | * | * | * | * |  | * | * | 8 |  |
| Ghanta, 2017 |  |  | * | * | * | * | * |  |  | 5 |  |
| Girard, 2009 | * | * | * | * | * | * | * | * | * | 9 |  |
| Habib, 2005 | * | * | * |  | * | * |  | * | * | 7 |  |
| Hakala, 2002 | * | * | * | * | * | * | * | * | * | 9 |  |
| Lee, 2018 | * | * | * | * | * | * | * |  |  | 7 |  |
| Ivanovic, 2014 | * | * | * | * | * | * | * | * | * | 9 |  |
| Kitahara, 2017 | * | * | * | * | * | * |  | * | * | 8 |  |
| Kuduvallia, 2002 | * | * | * | * | * | * | * | * | * | 9 |  |
| Moulton, 1996 | * | * | * |  | * | * |  | * | * | 7 |  |
| Melduni, 2011 | * | * | * | * | * | * | * | * | * | 9 |  |
| Omer, 2016 | * | * | * | * | * | * | * | * | * | 9 |  |
| Pan, 2006 | * | * | * |  | * | * |  | * | * | 7 |  |
| Perrier, 2016 | * | * | * | * |  |  | * | * | * | 7 |  |
| Reeves, 2003 | * | * | * |  | * | * | * |  | * | 7 |  |
| Stamou, 2011 | * | * | * |  | * | * |  | * | * | 7 |  |
| Sun, 2011 | * | * | * | * | * | * | * | * | * | 9 |  |
| Stefàno, 2020 | * | * | * | * | * | * | * | * |  | 8 |  |
| Tosello, 2015 | * | * | * | * |  |  | * | * | * | 7 |  |
| Tadic M, 2011 | * | * | * | * | * | * | * | * | * | 9 |  |
| Wong, 2015 | * | * | * | * |  | * | * | * |  | 7 |  |
| Yap, 2007 | * | * | * | * | * | * |  | * | * | 8 |  |
| Zacharias, 2005 | * | * | * | * | * | * | * |  |  | 7 |  |

1. Representativeness of the exposed cohort.
2. Selection of the non-exposed cohort.
3. Ascertainment of exposure.
4. Demonstration that outcome of interest was not present at start of study.
5. Comparability of cohorts on the basis of the design or analysis (adjusted for age).
6. Comparability of cohorts on the basis of the design or analysis (adjusted for any other factor).
7. Assessment of outcome.
8. Was follow-up long enough for outcomes to occur. (>5 days).
9. Adequacy of follow-up of cohorts.

**Table S5:** Relative risks between BMI and atrial fibrillation incident after cardiac operation, from the nonlinear dose-response analysis

| **Outcome**  **BMI (kg/m2)** | Atrial fibrillation after cardiac operation |
| --- | --- |
|  | RR (95% CI) |
| 22 | Ref |
| 25 | 1.03 (0.99-1.06) |
| 28 | 1.06 (0.99-1.13) |
| 30 | 1.07 (1.00-1.15) |
| 32 | 1.12 (1.02-1.23) |
| 34 | 1.16 (1.05-1.28) |
| 37 | 1.23 (1.08-1.41) |
| 39 | 1.29 (1.10-1.52) |
| 42 | 1.39 (1.12-1.73) |
| 44 | 1.46 (1.12-1.89) |
| 49 | 1.65 (1.13-2.42) |
| 52 | 1.76 (1.13-2.73) |
| *P*_nonlinearity_ | 0.44 |

Abbreviation: BMI, body mass index; RR, relative risk.

**SUPPLEMENTAL FIGURES**


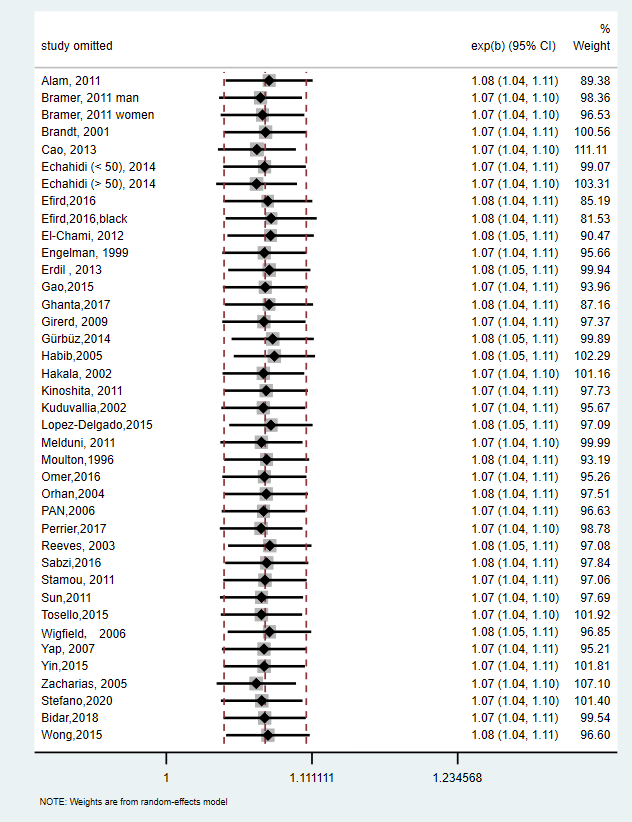


**Figure S1:** Sensitive analysis of BMI and POAF by omitting one study at each time, per 5 unit increased in BMI, dose-response analysis

Abbreviations: BMI = body mass index; SE=standard error; CI=confidence interval; POAF: atrial fibrillation after cardiac operation


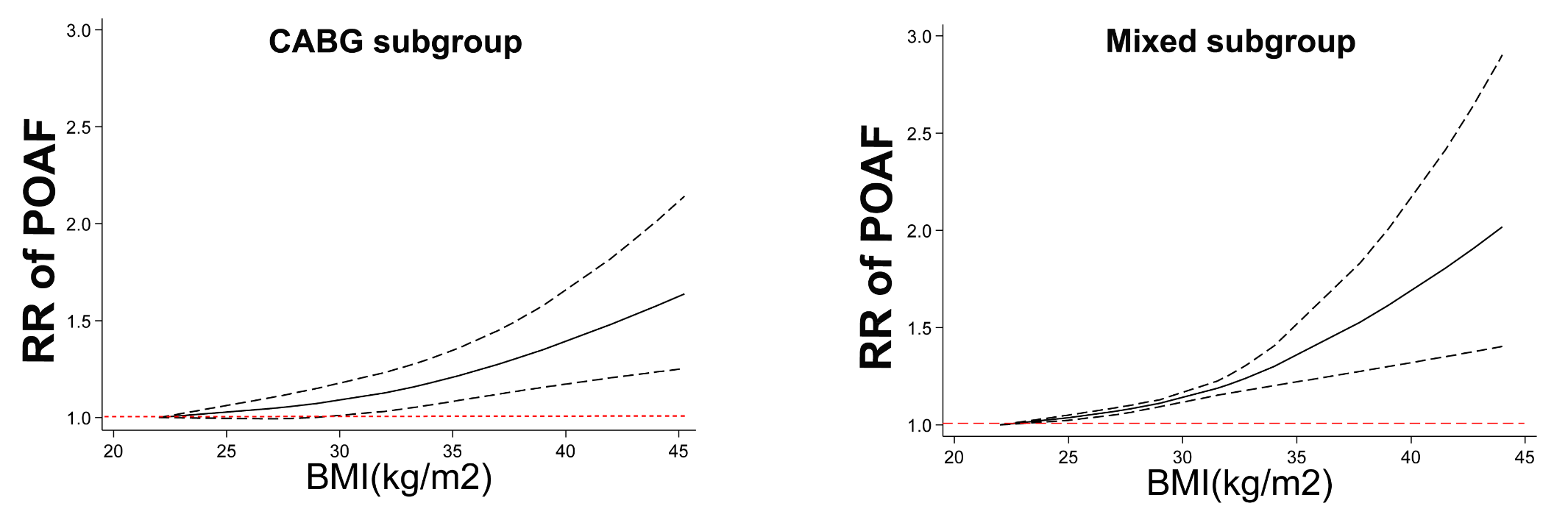


**Figure S2. Body mass index and POAF stratified by types of cardiac surgery and nonlinear exposure-effect analysis. The solid and dashed lines represent the estimated relative risk and the 95% confidence interval, respectively. A: coronary artery bypass graft; B: combined cardiac surgery.**

POAF: postoperative atrial fibrillation after cardiac surgery


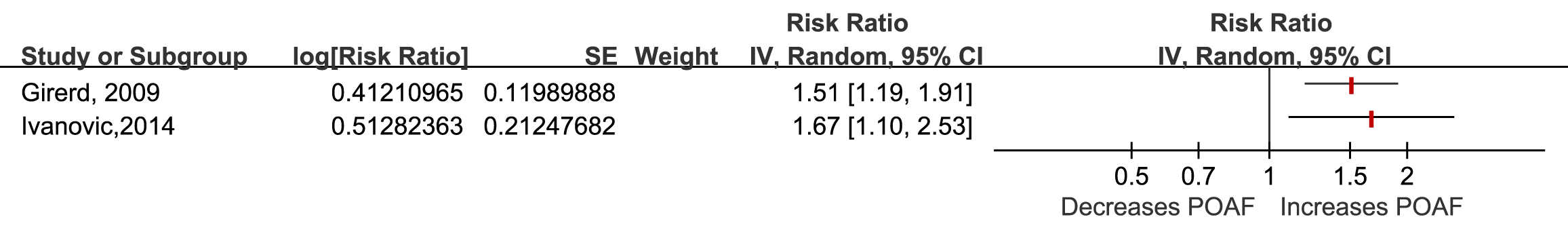


**Figure S3.** **Forest plot of the impact of waist obesity on POAF.**

POAF: postoperative atrial fibrillation after cardiac surgery


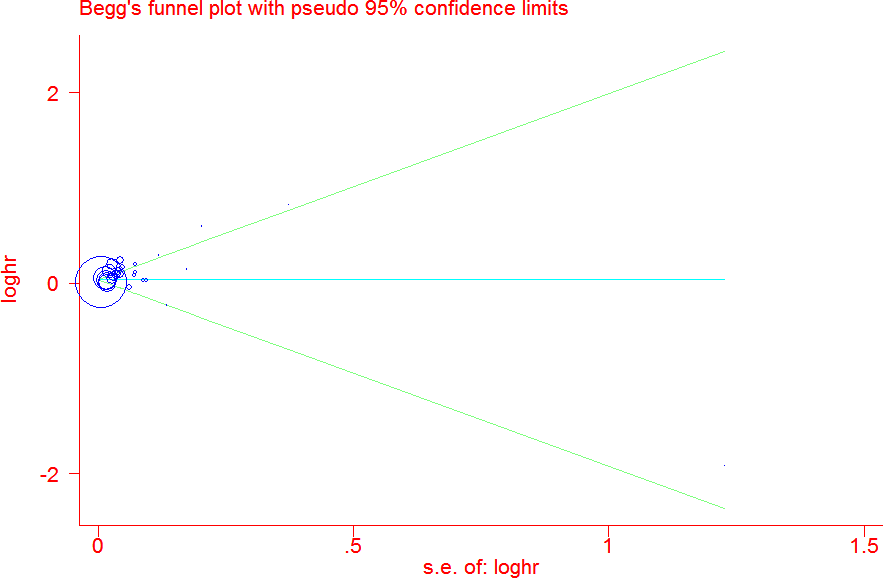


**Figure S4:** Publication bias analysis of the association between BMI and POAF, Begg’s test(P=0.73)

Abbreviations: BMI = body mass index; SE=standard error; POAF: atrial fibrillation after cardiac operation


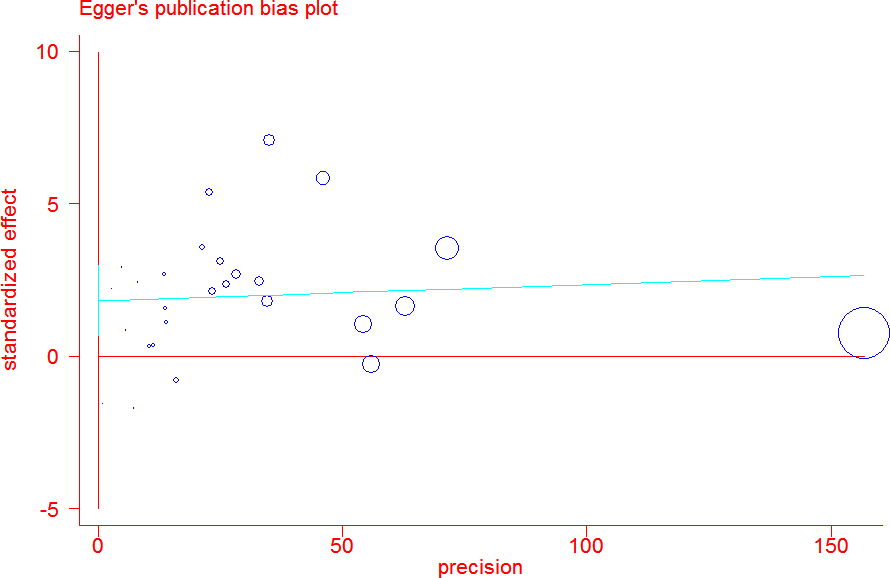


**Figure S5:** Publication bias analysis of the association between BMI and POAF, Egger’s test (P=0.42)

Abbreviations: BMI = body mass index; POAF: atrial fibrillation after cardiac operation


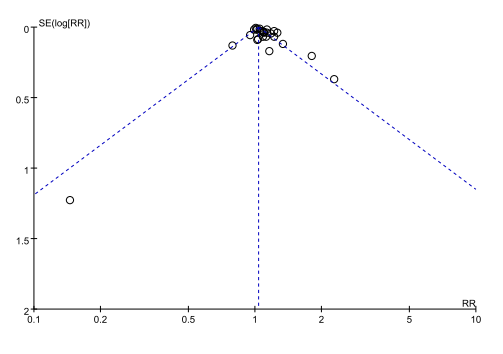


**Figure S6:** Publication bias analysis of the association between BMI and POAF, Funnel plot.

Abbreviations: BMI = body mass index; SE=standard error; POAF: atrial fibrillation after cardiac operation
